# An exact approach for studying cargo transport by an ensemble of molecular motors

**DOI:** 10.1186/2046-1682-6-14

**Published:** 2013-11-16

**Authors:** Donatello Materassi, Subhrajit Roychowdhury, Thomas Hays, Murti Salapaka

**Affiliations:** 1 Laboratory for Information and Decision Systems, Massachussets Institute of Technology, 77 Massachusetts Avenue Cambridge, MA 02139, USA; 2 Department of Electrical and Computer Engineering, University of Minnesota 200 Union Street SE, Minneapolis, MN 55455, USA; 3 Department of Genetics, Cell Biology, and Development, University of Minnesota, 321 Church Street SE, Minneapolis, MN 55455, USA

**Keywords:** Molecular motors, Rare event detection, Markov models

## Abstract

**Background:**

Intracellular transport is crucial for many cellular processes where a large fraction of the cargo is transferred by motor-proteins over a network of microtubules. Malfunctions in the transport mechanism underlie a number of medical maladies.

Existing methods for studying how motor-proteins coordinate the transfer of a shared cargo over a microtubule are either analytical or are based on Monte-Carlo simulations. Approaches that yield analytical results, while providing unique insights into transport mechanism, make simplifying assumptions, where a detailed characterization of important transport modalities is difficult to reach. On the other hand, Monte-Carlo based simulations can incorporate detailed characteristics of the transport mechanism; however, the quality of the results depend on the number and quality of simulation runs used in arriving at results. Here, for example, it is difficult to simulate and study rare-events that can trigger abnormalities in transport.

**Results:**

In this article, a semi-analytical methodology that determines the probability distribution function of motor-protein behavior in an exact manner is developed. The method utilizes a finite-dimensional projection of the underlying infinite-dimensional Markov model, which retains the Markov property, and enables the detailed and exact determination of motor configurations, from which meaningful inferences on transport characteristics of the original model can be derived.

**Conclusions:**

Under this novel probabilistic approach new insights about the mechanisms of action of these proteins are found, suggesting hypothesis about their behavior and driving the design and realization of new experiments.

The advantages provided in accuracy and efficiency make it possible to detect rare events in the motor protein dynamics, that could otherwise pass undetected using standard simulation methods. In this respect, the model has allowed to provide a possible explanation for possible mechanisms under which motor proteins could coordinate their motion.

## Background

The behavior of motor proteins is relatively well characterized when one motor protein is involved in the transport of a cargo. Indeed, it is possible to monitor the motion of a single molecular motor under highly tunable experimental conditions and obtain measurements with sufficiently accurate spatial and time resolution [1-3]. The resulting experimental data has led to many theoretical descriptions of motor-protein mechanisms which take into account the complex mechanochemical processes involved and yield insights into transitions between the multiple conformational states possible [4].

*In vivo*, often, an ensemble of molecular motors is responsible for the transport of a common cargo [5,6]. *In vitro* and simulation studies where multiple motors are involved in transport have provided unique insights into features of a common cargo being transported by many motors (see for example, [7,8]).

The dynamics when multiple motors transport cargo can be considerably more involved where a number of significant questions remain open. For example, it is not yet clear when and if motors synchronize their behavior, whether they move independently and whether they are antagonistically engaged in a “tug-of-war” [6,9]. Despite major improvements in instrumentation and techniques, understanding behavior of multiple coupled motors remains extremely challenging. The main difficulty is the substantially higher spatial and temporal resolution needs imposed by the fractional motion of the cargo and the increased number of possible transitions between conformational states [8,10]; possibilities introduced by the multiplicity of motors carrying a single cargo.

The available detailed characterization of how single motors transport cargo can be leveraged to develop models that describe how multiple-motors coordinate the motion of a common cargo. Indeed, using single molecule experimental data, accurate descriptions on the probability that a motor takes a step and its dependence on environmental factors such as temperature and ATP concentration, are reported in [11-13]. Similar estimates on the attachment and detachment rates of molecular motors to and from a microtubule can be found in [13-15]. A model that describes how multiple motors carry a common cargo can be obtained by using the information on single motor protein behavior and by introducing the coupling of the individual motor-proteins via the dynamics of the shared common cargo. Using Monte-Carlo simulations on such a model [7], reported novel insights into the behavior of kinesin motors, such as, a smaller velocity of transport of cargo when carried by multiple motors as opposed to a single one, and a dependence of the expected run-length on the stiffness of the motor linkage. While Monte-Carlo techniques form an important set of tools, they involve a trade-off between the accuracy desired and the computational effort needed. As a consequence, important features of the dynamics, especially if associated with rare events, can be missed. This aspect takes particular significance in the study of biological systems, where pathological behaviors are caused or triggered by events which are improbable under normal conditions but occur with significant adverse impact.

Existing approaches have utilized models with simplifying assumptions that can be treated analytically or semi-analytically in order to understand the basic features of the coordinated motion of motor proteins. For example, in [16] mean-field theory is applied for analyzing large ensembles of motors, whereas, in [17] the cooperative transport of cargo realized by two motor proteins is studied in order to identify distinct operational regimes. In [14] apart from providing estimates of attachment and detachment rates of motors to microtubules, analytical dependence of run-length on the number of motors involved in the transport of a common cargo is obtained.

In this article, we present a general methodology which determines the probability distribution function of various motor behaviors. This different approach provides several advantages over Monte-Carlo simulation based methods. In our method the probabilities of outcomes are determined exactly, unlike Monte-Carlo simulation based methods; however, our method does not sacrifice the detailed description of the system possible with Monte-Carlo simulations. Our strategy is particularly well suited for characterizing rare-events that take prohibitive number of simulations in a Monte-Carlo setting. Moreover, in the new framework, delineation of the detailed causes of an observed functionality is straightforward (which involves a simple step of identifying states that are associated with the observation and analyzing these states). At the same time, our model has a high level of accuracy and detail. Compared with other analytical studies, such as the ones previously reported, a larger number of motors can be studied. In [18] and [17] the study is limited to only two motors and certain simplifying assumptions are often made (i.e. the aggregation of microstates with same energy in [18]). In [18] a stochastic model that takes into account only the number of motors engaged on the microtubule is adopted in order to understand the level of coupling among two motor proteins carrying a common cargo. In [16] groups of more than two motor proteins are studied. Related work [14] alluded to earlier analyzes the run-length, average velocity, steady state distribution of bound motors and effects of load force on velocities. In both, the mean-field approach of [16] and the approach in [14], the proteins are not individually modeled anymore (for example, it is assumed that the load is equally shared on all the engaged motors). Under the methodology described in this paper, each motor is individually modeled and analytical or semi-analytical results can still be provided. Thus, more accurate conclusions on how the interaction between multiple-motors affects a transportation modality can be reached.

The article develops a Markov model, where the number of motors at any particular location on the microtubule lattice form states, and such a state determines the location of the common shared cargo. Here the transition probabilities between states can be derived from studies on single motor-protein based transport. The physics of the system is utilized to project the resulting infinite dimensional model onto a finite dimensional one. We show that the finite dimensional model, apart from the benefit of increased computational tractability, has other important features such as the existence of a unique steady-state probability distribution. Furthermore, we demonstrate that the probability distribution of the projected model can be used to answer most of the biologically relevant queries on transport modality. In particular, probabilities of rare events and the related mechanisms can be unraveled. The capabilities of the methodology are tested with existing data and via extensive Monte-Carlo simulations. These features can significantly ease the computational burden as well as provide unique insights into transport modalities.

## Methods

Here we provide a methodology for analyzing the dynamics of an ensemble of motor proteins carrying a single cargo on a microtubule lattice. Each individual motor behavior is described stochastically: it can detach or attach to the microtubule and take steps on the filament according to prescribed probabilities that are governed by specified transition rates. The derived stochastic model provides an intuitive representation of the physical system, but, being infinite-dimensional, is not tractable and provides no general guarantees on the existence of stationary steady behavior. This impasse is overcome by building an alternative, and effective, Markov model with the advantage of being described by a *finite* number of states. In this model only the information pertinent to the relative configuration of the motor-proteins is incorporated where the relative positions of motor proteins with respect to each other determine the state. The evaluation of the probability distribution for all these possible arrangements can be determined by computing the exponential of a matrix with a dimension that is dependent on the number of arrangements. We show that the number of states does not become excessively large and that the solution via matrix exponential is viable, allowing a direct way to compute the probability distribution of motor arrangements. Furthermore we show how quantities of interest such as, average cargo run-length, average number of engaged motors and average speed of the cargo can be derived, from the determined probability distribution on the relative configurations.

We instantiate the methodology to the case where cargoes are transported by multiple kinesin motors. Despite being specific to these molecules, most of these strategies can be extended or adapted to other classes of motor proteins and also to model a cargo transported by multiple species of proteins, as well.

### Description of the system and main modeling assumptions

**Figure 1:**
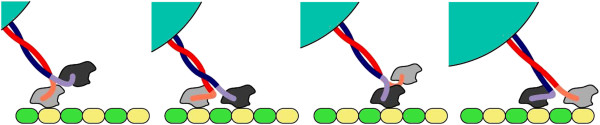
**Four stages describing the processive motion of a single molecular motor on a microtubule.** The microtubule is represented as a sequence of equally spaced locations (yellow and green color). The motor protein consists of two heads represented in light and dark gray and a linkage depicted as two intertwined filaments (red and blue color). In the first stage the light gray head is connected to the microtubule. In the second stage the dark gray head binds as well. The hydrolization of a molecule of ATP properls the light gray head forward (third stage). In the forth stage both heads are again bound to the microtubule but this time the dark gray one is located behind. By repeating these stages, the motor protein transports the cargo particle (cyan color).

The motion of a motor occurs by discrete steps on a microtubule. Their heads move forward by hydrolyzing ATP and producing shear forces against specific binding sites that are equally spaced (see Figure 1).

Every motor of the ensemble is bound to the cargo molecule via a flexible linkage. We assume that the linkage has a known rest length *l*_0_, which behaves like an elastic spring when stretched, and offers no resistance when compressed [7]. In particular, the exerted force *F*, as a function of its length *l*, is expressed as

(1) $$\begin{array}{l} F(l)=\left\{\begin{array}{cc} k_{\text{el}}(l+l_{0}) & \text{if}\;l\leq -l_{0}\\ 0 & \text{if}\;|l|<l_{0}\\ k_{\text{el}}(l-l_{0}) & \text{if}\;l\geq l_{0}, \end{array}\right) \end{array}$$

where *k*_*el*_ is the stiffness of the linkage. If the linkage is stretched beyond a certain stalling force *F*_*s*_, the motor can not take any forward step. We remark that *F*_*s*_ is typically measured in order to quantify the number of motors that are actively pulling a cargo. Backward steps are neglected in the model and the motors are irreversibly bound to the cargo particle. A motor head that is attached to the microtubule has a certain chance of detaching from it, while a motor head that is not attached has a certain chance of binding to the microtubule. An unbound motor-protein can bind to the microtubule at a location only when it is within a distance *l*_0_ of the cargo. Thus a floating motor binds to the microtubule without stretching its linkage. The cargo is subjected to a constant load *F*_*load*_ that opposes the motor motion. The cargo position is described in probabilistic terms by a Gaussian distribution with variance *σ*_*th*_ and truncated on the interval [−3*σ*_*th*_,3*σ*_*th*_]. The mean position of the cargo *x*_*eq*_ is the equilibrium position determined by the load *F*_*load*_ on the cargo and forces exerted by the motors through their linkages. The effect of thermal fluctuations is incorporated into the probabilities of cargo position by determining the variance parameter *σ*_*th*_ of the cargo position in a steady state situation.

When a motor steps forward or detaches, the probability distribution of the position of the cargo is assumed to reach a new distribution with negligible transient. Thus we assume that the time scale of the cargo dynamics is much faster than the rate at which motor configurations change. The system is assumed to be spatially invariant: its stochastic behavior does not change if the motor ensemble and the cargo shift to a new position along the microtubule. Finally, if, at any time, there are no motors engaged with the microtubule, the cargo is assumed to be “lost” which forms the stopping criterion for the stochastic model.

**Figure 2:**
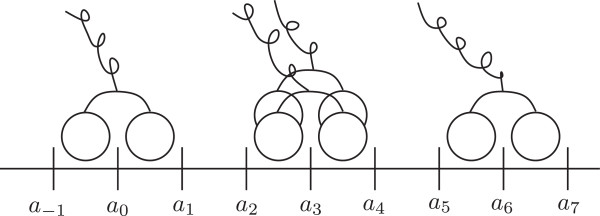
**Schematic representation of the configuration of an ensemble of motors.** The microtubule is represented as a bi-infinite filament with equally spaced location $\left\{\ldots ,a_{-1},a_{0},a_{1},\ldots a_{7},\ldots \;\right\}$. The rear-guard motor is engaged at location *a*_0_, two are engaged in configuration *a*_3_ and a fourth one is engage at location *a*_6_.

The microtubule is modeled as a sequence of equally spaced locations *a*_*k*_=*a*_0_+*kd*_*s*_ where *a*_*k*_ represents the linear position of the *k*-th location, *k* is an integer index and *d*_*s*_ is the periodicity of the filament (in the case of microtubules *d*_*s*_=8 *nm*). We assume that $\bar{m}$ motors constitute the ensemble. They are all permanently bound to the cargo particle while they can be engaged or not with the microtubule. We represent the locations of motors with a bi-infinite sequence of natural numbers $Z:={\left\{z_{k}\right\}}_{k\in I}$ where the *z*_*k*_ are the number of motors engaged on the microtubule at the location *a*_*k*_ and  is the set of integer numbers. This bi-infinite sequence *Z* provides the *absolute configuration* of the motors on the microtubule lattice (see Figure 2).

In the model, it is assumed that multiple proteins could share the same location on the microtubule, even though the motor proteins actually bind to physically different areas of the cargo macromolecule. The motivation and justification for this assumption are provided later.

We denote the set of all absolute configurations as **Z**. For any absolute configuration *Z*, we define the right-shift operator *ρ* that moves all the terms *z*_*k*_ by one place to the right. In a similar manner we define the left-shift operator *ρ*^−1^ and generalize the notation to *ρ*^*α*^ for a shift by *α* places. For a fixed value of *F*_*load*_>0, the mean cargo position *x*_*eq*_ is a function of the absolute configuration *Z*, that is *x*_*eq*_=*x*_*eq*_(*Z*). There are only three possible transitions from one configuration *Z* to another $Z^{\prime }$: a motor can step forward to the next location; if attached then it can detach from the microtubule; and if unattached it can attach to the microtubule. We represent the transition from an absolute configuration *Z* to another absolute configuration $Z^{\prime }$ as $Z\to Z^{\prime }=Z+R$, where *R* is a suitable sequence that characterizes the specific transition. For example, in the case of a motor at location *a*_*k*_ stepping forward, the transition is represented as follows

$$\begin{array}{l} \;Z=\left(\begin{array}{c} \vdots \\ z_{k}\\ z_{k+1}\\ \vdots \end{array}\right)\overset{\text{STEP}}{\to }\left(\begin{array}{c} \vdots \\ z_{k}\\ z_{k+1}\\ \vdots \end{array}\right)+\left(\begin{array}{c} \vdots \\ -1\\ +1\\ \vdots \end{array}\right)=Z+R_{k}^{\left(\text{step}\right)}. \end{array}$$

Analogously, for a attachment/detachment transition at location *a*_*k*_, we have

$$\begin{array}{l} \;Z=\left(\begin{array}{c} \vdots \\ z_{k}\\ z_{k+1}\\ \vdots \end{array}\right)\overset{\text{ATT}/\text{DET}}{\to }\left(\begin{array}{c} \vdots \\ z_{k}\\ z_{k+1}\\ \vdots \end{array}\right)+\left(\begin{array}{c} \vdots \\ \pm 1\\ 0\\ \vdots \end{array}\right)=Z\pm R_{k}^{\left(\text{att}\right)} \end{array}$$

where the plus sign (+) is for the attachment transition and the minus sign (−) is for the detachment transition. The sequences $R_{k}^{\left(\text{step}\right)}$ and $R_{k}^{\left(\text{att}\right)}$ represent the change in number of motors from the starting configuration *Z* to the ending configuration $Z^{\prime }$. Assuming that the probability rate of the transition Z→Z+R is known and is given by *λ*_*a* *b* *s*_(*Z*+*R*,*Z*), it is possible to define an infinite dimensional Markov model, analogous to the ones described in [19,20]. Here $\lambda_{\text{abs}}(Z^{\prime },Z)\mathrm{\Delta t}$ denotes the probability that the absolute configuration is $Z^{\prime }$ at time *t*+*Δt* given that it was *Z* at time *t*. Implicit is the assumption that *λ*_*a* *b* *s*_ does not depend on *t*. It follows that, given an initial time *t*_0_ and an initial state $\bar{Z}$, for *t*≥*t*_0_, $P_{\text{abs}}(Z,t|\bar{Z},t_{0})$, the probability of the absolute configuration being equal to *Z* at time *t* given that it was equal to $\bar{Z}$ at *t*_0_ satisfies the Master Equation

(2) $$\begin{array}{ll} \frac{\partial }{\mathrm{\partial t}}P_{\text{abs}}(Z,t|\bar{Z},t_{0}) & =-P_{\text{abs}}(Z,t|\bar{Z},t_{0})\underset{Z^{\prime }\in Z}{\sum }\lambda_{\text{abs}}(Z^{\prime },Z)\\ & \;+\underset{Z^{\prime }\in Z}{\sum }\lambda_{\text{abs}}(Z,Z^{\prime })P_{\text{abs}}(Z^{\prime },t|\bar{Z},t_{0}), \end{array}$$

that represents the conservation law of the probability measure. We will drop the conditioning on the initial absolute configuration being $\bar{Z}$ at time *t*_0_ and assume that all probabilities described below are implicitly conditioned on

$$(\bar{Z},t_{0}).$$

We also observe that the spatial invariance hypothesis translates into an immediate condition on the transition rates, namely that $\lambda_{\text{abs}}(Z^{\prime },Z)=\lambda_{\text{abs}}(\rho^{\alpha }Z^{\prime },\rho^{\alpha }Z)$ for any integer *α*. This condition, along with the presence of a stalling force for the motors, is used to arrive at an effective finite-dimensional Markov model.

### Derivation of an effective finite-dimensional Markov model

The representation of an ensemble of motors as a bi-infinite sequence allows one to describe the system in a rather intuitive manner and highlights the similarities with a Gillespie model for the purpose of stochastic simulations [19,20]. However, such a model is ill-suited for an exact analysis because of its infinite dimension. A finite dimensional model can be obtained by aggregating (or projecting) states of the infinite dimensional model into “macro-states”. In general, this approach leads to the loss of the Markov property. However, in the following we provide a projection of the infinite states of the original model on a finite set in such a way that the Markov property is preserved. This allows us to pursue an exact analysis and determine explicit formulas for the computation of biologically relevant quantities.

**Figure 3:**
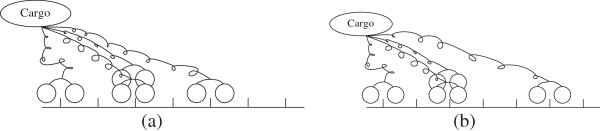
**The string representation for the arrangement of four motors in (a) is “ *M *** **|| *M M *** **|| *M *” and, after the leading motor has stepped, the representation changes into “ *M *** **|| *M M *** **||| *M *”, as depicted in (b).**

To arrive at the relative configuration description, we represent the arrangement of motors using strings of two symbols. The empty string Ø refers to the case where there are no motors engaged on the microtubule (loss of the cargo). The engaged motor that lags behind all the other motors is the “rear-guard” motor and serves as a reference. Starting with the rear-guard motor we write a symbol (’M’) for a motor in each location and use a separator (’ |’) to distinguish distinct locations. As an example, the configuration of four motors shown in Figure 3(a) is represented as “ *M*||*MM*||*M*” and, after the leading motor has stepped, the representation changes to “ *M*||*MM*|||*M*” (see Figure 3(b)).

This intuitive string representation provides the *relative configuration* which characterizes how various motors carrying the cargo are positioned with respect to each other.

We make the following observations: 

• Strings representing relative configurations can have arbitrary length.

• Two different absolute configurations of the motors, $Z^{\prime }$ and *Z*, on the microtubule may have the same relative configuration if *Z* is a “shifted version” of $Z^{\prime }.$ Two absolute configurations have the same relative configuration, if and only if the relative distances among the engaged motors of the ensemble are the same. This defines a class of equivalence on absolute configurations: two absolute configurations belong to the same equivalence class if both have the same relative configuration.

• From a relative configuration we can obtain the relative positions of the motors, but not their absolute positions on the microtubule lattice.

Consider the following assumptions on the model, 

1. An ensemble contains $\bar{m}$ molecular motors (which is the number of motors attached to the cargo)

2. Motor linkages are elastic springs with constant *k*_*el*_ and rest length *l*_0_

3. There is constant load *F*_*load*_ on the cargo

4. The stalling force is *F*_*s*_

5. An unattached motor can attach to the microtubule only to locations that are within distance *l*_0_ from the cargo center of mass (the attachment occurs a locations that are close enough not to stretch the linkage)

6. All motors are attached at the same location on the cargo and multiple motors can share the same microtubule location.

The last assumption is introduced for the following reason. From a mathematical perspective, there is no loss of generality on assuming that all molecular motors are bound to the same cargo location. Indeed it is possible to apply a coordinate change to each motor’s position whereby all motors are attached at the same location on the cargo. With this assumption we have to allow for multiple motors to be attached to the same microtubule location, as, identically stretched motors that are physically attached to the cargo at different locations get mapped, in the new coordinate system, as being attached at the same location on the cargo and the microtubule.

Under the above assumptions we have established that the maximum distance (expressed in number of locations on the microtubule) between the vanguard motor and rearguard motor is bounded by

(3) $$n:=\left\lceil \max\left\{\frac{\bar{m}F_{s}-F_{\text{load}}}{k_{\text{el}}d_{s}}+1,\frac{F_{\text{load}}}{k_{\text{el}}d_{s}}\right\}+\frac{2l_{0}}{\text{ds}}+\frac{6\sigma_{\text{th}}}{d_{s}}\right\rceil$$

where ⌈·⌉ represents the ceiling function. The main intuition on how the various factors in (3) contribute follows from the stall condition on the motors, where, a motor cannot step forward if it experiences a force greater than the stall force *F*_*s*_. For example, $\frac{\bar{m}F_{s}-F_{\text{load}}}{k_{\text{el}}d_{s}}+1$ is the maximum distance between the rearguard and vanguard motor possible, beyond which motors stall, $\frac{6\sigma_{\text{th}}}{d_{s}}$ accounts for the thermal noise contribution, whereas, $\frac{2\ell_{0}}{d_{s}}$ accounts for the possibility that motors are within a distance 2*ℓ*_0_ where the motors are not stretched at all.

We will establish the above result precisely when there is at least one motor opposing the motion of the cargo in the absence of thermal noise (the other cases are less involved and are based on similar arguments). Without any loss of generality, let us consider the cargo equilibrium position *x*_*eq*_=0. Let positions of the motors that assist the motion be *x*_*v*_, *x*_*v*−1_,…,*x*_1_ with *x*_*v*_≥*x*_*v*−1_≥…≥*x*_1_≥*ℓ*_0_ and the corresponding forces exerted by motors be *Fv*+, *Fv*−1+,…, *F*1+. Similarly let the positions of motors opposing the motion be given by −*y*_1_, −*y*_2_, …−*y*_*r*_ with *y*_*r*_≥*y*_*r*−1_≥…≥*y*_1_≥*ℓ*_0_>0 and the corresponding forces on the cargo be *F*1−, *F*2−,…, *Fr*− (these forces oppose the motion of the cargo). Note that *Fj*+=*k*_*el*_(*x*_*j*_−*ℓ*_0_) and *Fj*−=*k*_*el*_(*y*_*j*_−*ℓ*_0_) and the separation *S* (which we term *extent*) between the vanguard and rearguard motors is *x*_*v*_+*y*_*r*_. We also note that *Fr*−=*k*_*el*_(*y*_*r*_−*ℓ*_0_)=*k*_*el*_(*y*_*r*_+*x*_*v*_−*x*_*v*_−*ℓ*_0_)=*k*_*el*_*S*−*Fv*+−2*k*_*el*_*ℓ*_0_. Under equilibrium it follows that

$$\;\begin{array}{ll} F_{\text{load}} & =F_{v}^{+}+\overset{v-1}{\underset{i=1}{\sum }}F_{i}^{+}-\overset{r-1}{\underset{j=1}{\sum }}F_{j}^{-}-F_{r}^{-}\\ & =F_{v}^{+}+\overset{v-1}{\underset{i=1}{\sum }}F_{i}^{+}-\overset{r-1}{\underset{j=1}{\sum }}F_{j}^{-}-k_{\text{el}}S+F_{v}^{+}+2k_{\text{el}}\ell_{0} \end{array}$$

and thus

$$\begin{array}{c} k_{\text{el}}S=2F_{v}^{+}+\overset{v-1}{\underset{i=1}{\sum }}F_{i}^{+}-\overset{r-1}{\underset{j=1}{\sum }}F_{j}^{-}+2k_{\text{el}}\ell_{0}-F_{\text{load}}. \end{array}$$

Now suppose that the vanguard motor (and therefore all motors) is not stalled (that is *Fv*+≤*F*_*s*_) then it follows that

$$\begin{array}{l} k_{\text{el}}S=2F_{v}^{+}+\overset{v-1}{\underset{i=1}{\sum }}F_{i}^{+}-\overset{r-1}{\underset{j=1}{\sum }}F_{j}^{-}+2k_{l}\ell_{0}-F_{\text{load}}\\ \leq 2F_{v}^{+}+\overset{v-1}{\underset{i=1}{\sum }}F_{i}^{+}+2k_{\text{el}}\ell_{0}-F_{\text{load}}\\ \leq \bar{m}F_{s}+2k_{\text{el}}\ell_{0}-F_{\text{load}} \end{array}$$

Let $s^{\left(\text{max}\right)}:=\frac{\bar{m}F_{s}-F_{\text{load}}}{k_{\text{el}}}+2\ell_{0}+d_{s}$. It follows that if none of the motors are stalled then the extent *S*≤*s*^(*m* *a* *x*)^−*d*_*s*_.

Now we can assert that if the extent was less than or equal to *s*^(*m* *a* *x*)^ then for any subsequent change in the configuration, the extent will still remain less than *s*^(*m* *a* *x*)^. Indeed, consider the case where the current configuration is such that the extent *S*≤*s*^(*m* *a* *x*)^. There are two possibilities for the current configuration (a) the vanguard motor is stalled in which case the extent can only decrease in any subsequent change in the configuration as the vanguard motor cannot step forward and the rearguard motor cannot step backwards (b) the vanguard motor in the current configuration is not under stall in which case the extent *S*≤*s*^(*m* *a* *x*)^−*d*_*s*_. In any subsequent change the only means to increase the extent is when the vanguard motor takes a step with a step-size *d*_*s*_ where the extent still remains bounded by *s*^(*m* *a* *x*)^. Thus we have shown that if the extent of an absolute configuration is smaller than a bound *s*^(*m* *a* *x*)^ then for all future configurations this bound is respected.

Using combinatorial calculus, it follows that the number *N* of possible relative configurations is

(4) $$\begin{array}{l} N=1+\overset{\bar{m}}{\underset{m=1}{\sum }}\frac{(n+m-2)!}{(n-1)!(m-1)!}. \end{array}$$

**Figure 4:**
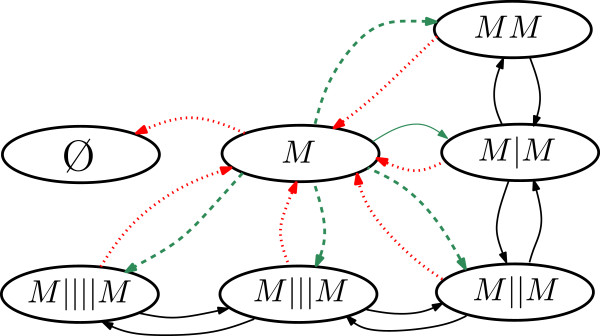
**The graph that represents the symbolic dynamics in the case of**$\bar{m}=2$** with the simplifying additional assumption that the two motors are never at a distance larger than four locations from each other.** A red arrow represents a detachment, a green arrow represents an attachment and a black arrow represents a forward step of one of the two motors. As it can be seen the undirected version of this graph is connected.

Each bi-infinte sequence *Z* that codes the absolute configuration, determines in a unique way a string representation that codes its relative configuration, and thus transitions Z→Z+R of the infinite dimensional model determine transitions from one string representation to another. In Figure 4 we provide an example of a graph representing the symbolic dynamics in the case of $\bar{m}=2$ where the maximum distance between the vanguard motor and the rearguard motor is four locations. A red-dotted arrow is used to represent a detachment transition, a green-dashed arrow represents an attachment event, and a black-solid arrow represents a forward step of one of the two motors.

Notice that physically different simple events can give rise to the same transition in the symbolic dynamics of the strings. For example, from the string *M*|*M* it is possible to reach the string *M* because of a detachment of either the vanguard or the rearguard motor.What has been achieved so far is a projection of the model dynamics from the set of absolute configurations (*Z*) with infinitely many elements to a space of relative configurations (*σ*) with finitely many configurations. We denote the projector operator as *σ*=*Π*^(*e*)^(*Z*) where the absolute configuration *Z* has a relative configuration *σ*.

Also, we define the set Z(σ) of all absolute configurations with the same relative configuration *σ*.

$$\begin{array}{l} Z(\sigma ):=\{Z|\Pi^{\left(e\right)}(Z)=\sigma \} \end{array}$$

In general projections do not preserve the Markov property of a model. However, in this case, we can show that the dynamics on the string space still maintains the Markov property. More importantly, the transition rate *λ*_*rel*_(*σ*^′^,*σ*) from one string *σ* to another string *σ*^′^ can be meaningfully defined and can be computed from the knowledge of the rates $\lambda_{\text{abs}}(Z^{\prime },Z)$ of the original Gillespie model. We now determine $\lambda_{\text{rel}}(\sigma^{\prime },\sigma )$.

For small *Δt*, note that the probability that the absolute configuration is $Z^{\prime }$ at time *t*+*Δt* given that it was at *Z* at time *t* is given by $P_{\text{abs}}(Z^{\prime },t+\mathrm{\Delta t}|Z,t)=\lambda_{\text{abs}}(Z^{\prime },Z)\mathrm{\Delta t}$. Similarly, let $P_{\text{rel}}(\sigma^{\prime },t+\mathrm{\Delta t}|\sigma ,t)=\lambda_{\text{rel}}(\sigma^{\prime },\sigma ,t)\mathrm{\Delta t}$ denote the probability that the relative configuration is at $\sigma^{\prime }$ at time *t*+*Δt* given that it was at *σ* at time *t*. We now derive the transition probabilities in the relative configuration space from the transition probabilities in the absolute configuration space. It is evident from Bayes’ rule that

$$\begin{array}{l} P_{\text{rel}}(\sigma^{\prime },t+\mathrm{\Delta t}|\sigma ,t)=P_{\text{rel}}(\sigma^{\prime },t+\mathrm{\Delta t},\sigma ,t)/P_{\text{rel}}(\sigma (t)). \end{array}$$

where $P_{\text{rel}}(\sigma^{\prime },t+\mathrm{\Delta t},\sigma ,t)$ is the probability that the relative configuration at time *t* is *σ* and is $\sigma^{\prime }$ at time *t*+*Δt*. $P_{\text{rel}}(\sigma^{\prime },t+\mathrm{\Delta t},\sigma ,t)$ can be obtained by summing over the probabilities $P_{\text{abs}}(K^{\prime },t+\mathrm{\Delta t},K,t)$ of all pairs of absolute configurations *K* and $K^{\prime }$ that have relative configurations *σ* at time *t* and $\sigma^{\prime }$ at time *t*+*Δt* respectively i.e.

$$\begin{array}{l} \;P_{\text{rel}}(\sigma^{\prime },t+\mathrm{\Delta t},\sigma ,t)=\;\underset{K\in Z(\sigma )}{\sum }\underset{K^{\prime }\in Z(\sigma^{\prime })}{\sum }P_{\text{abs}}(K^{\prime },t+\mathrm{\Delta t},K,t) \end{array}$$

and similarly it follows that $P_{\text{rel}}(\sigma (t))=\underset{K\in Z(\sigma )}{\sum }P_{\text{abs}}(K,t)$. Now, arbitrarily choose $Z^{\prime }$ and *Z* such that $\Pi^{\left(e\right)}(Z^{\prime })=\sigma^{\prime }$ and *Π*^(*e*)^(*Z*)=*σ*. From the translation invariance property it follows that $Z(\sigma )=\{\rho^{\alpha }Z:\alpha \in I\}$ and $Z(\sigma^{\prime })=\{\rho^{\beta }Z^{\prime }:\beta \in I\}$ where *ρ*^*α*^ denotes a shift by *α* positions along the microtubule and  denotes the set of integers. Thus, all absolute configurations with a relative configuration *σ* can be obtained by taking one absolute configuration *Z* with relative configuration *σ* and forming the set of all possible shifts of the one absolute configuration *Z*. Thus, it follows that

(5) $$\begin{array}{ll} \;P_{\text{rel}}(\sigma^{\prime },t+\mathrm{\Delta t}|\sigma ,t) & =P_{\text{rel}}(\sigma^{\prime },t+\mathrm{\Delta t},\sigma ,t)/P_{\text{rel}}(\sigma (t))\\ & =\frac{\underset{K\in Z(\sigma )}{\sum }\;\underset{K^{\prime }\in Z(\sigma^{\prime })\;}{\sum }P_{\text{abs}}(K^{\prime },t\;+\;\mathrm{\Delta t},K,t)}{\underset{K\in Z(\sigma )}{\sum }P_{\text{abs}}(K,t)}\\ & =\frac{1}{\underset{\alpha }{\sum }P_{\text{abs}}(\rho^{\alpha }Z,t)}\sum_{\alpha }\sum_{\beta }P_{\text{abs}}(\rho^{\beta }Z^{\prime },t\\ & \;+\mathrm{\Delta t},\rho^{\alpha }Z,t)\\ & =\frac{1}{\underset{\alpha }{\sum }P_{\text{abs}}(\rho^{\alpha }Z,t)}\sum_{\alpha }\sum_{\beta }P_{\text{abs}}(\rho^{\beta }Z^{\prime },t\\ & \;+\mathrm{\Delta t}|\rho^{\alpha }Z,t)P_{\text{abs}}(\rho^{\alpha }Z,t)\\ & =\frac{1}{\underset{\alpha }{\sum }P_{\text{abs}}(\rho^{\alpha }Z,t)}\sum_{\alpha }P_{\text{abs}}(\rho^{\alpha }Z,t)\\ & \;\times \underset{\beta }{\sum }P_{\text{abs}}(\rho^{\beta }Z^{\prime },t+\mathrm{\Delta t}|\rho^{\alpha }Z,t)\\ & =\frac{1}{\underset{\alpha }{\sum }P_{\text{abs}}(\rho^{\alpha }Z,t)}\sum_{\alpha }P_{\text{abs}}(\rho^{\alpha }Z,t)\\ & \;\times \sum_{\beta }P_{\text{abs}}(\rho^{\left(\beta -\alpha \right)}Z^{\prime },t+\mathrm{\Delta t}|Z,t)\\ & =\frac{1}{\underset{\alpha }{\sum }P_{\text{abs}}(\rho^{\alpha }Z,t)}\sum_{\alpha }P_{\text{abs}}(\rho^{\alpha }Z,t)\\ & \;\times \sum_{\beta }P_{\text{abs}}(\rho^{\beta }Z^{\prime },t+\mathrm{\Delta t}|Z,t)\\ & =\sum_{\beta }P_{\text{abs}}(\rho^{\beta }Z^{\prime },t+\mathrm{\Delta t}|Z,t)\\ & =\sum_{\beta }\lambda_{\text{abs}}(\rho^{\beta }Z^{\prime },Z)\mathrm{\Delta t}\\ & =\sum_{K^{\prime }\in Z(\sigma^{\prime })}\lambda_{\text{abs}}(K^{\prime },Z)\mathrm{\Delta t} \end{array}$$

where the first three equalities have been explained before, the fourth follows from Bayes’rule and the fifth is evident. The sixth equality uses translation invariance where the absolute configurations at *t* and *t*+*Δt* are both shifted by *ρ*^−*α*^, the seventh follows from the fact that the set $\left\{\rho^{\left(\beta -\alpha \right)}:\beta \in I\}=\{\rho^{\beta }:\beta \in I\right\}$ where *α* is fixed and *β* is any integer (with  denoting the set of integers).

Note that in Equation (5), *Z* was arbitrarily chosen such that *Π*^(*e*)^(*Z*)=*σ*. Thus, the relation must hold for every Z∈Z(σ), yielding

$$\begin{array}{cc} \;P_{\text{rel}}(\sigma^{\prime },t\;+\;\mathrm{\Delta t}|\sigma ,t)\;=\;\; & \underset{K^{\prime }\in Z(\sigma^{\prime })}{\sum }\lambda_{\text{abs}}(K^{\prime },Z)\mathrm{\Delta t}\;\text{for all}Z\in Z(\sigma ). \end{array}$$

Thus, we can write

$$\begin{array}{cc} P_{\text{rel}}(\sigma^{\prime },t+\mathrm{\Delta t}|\sigma ,t) & =\underset{K\in Z(\sigma )}{\min}\underset{K^{\prime }\in Z(\sigma^{\prime })}{\sum }\lambda_{\text{abs}}(K^{\prime },K)\Delta \end{array}$$

where the min operator has been introduced just to obtain a term that formally depends on *σ* and $\sigma^{\prime }$ only.

We can define the rate of transition from the relative configuration *σ* to a relative configuration $\sigma^{\prime }$ as $\lambda_{\sigma }(\sigma^{\prime },\sigma )$ where $P_{\text{rel}}(\sigma^{\prime },t+\mathrm{\Delta t}|\sigma ,t)=\lambda_{\text{rel}}(\sigma^{\prime },\sigma )\mathrm{\Delta t}$ with

(6) $$\lambda_{\text{rel}}(\sigma^{\prime },\sigma ):=\underset{K\in Z(\sigma )}{\min}\underset{K^{\prime }\in Z(\sigma^{\prime })}{\sum }\lambda_{\text{abs}}(K^{\prime },K).$$

The knowledge of the transition rates (6) can be exploited, using the Bayes’ rule and the law of total probability, to obtain

(7) $$\begin{array}{ll} \frac{\partial }{\mathrm{\partial t}}P_{\text{abs}}(\sigma ,t)= & -P_{\text{abs}}(\sigma ,t)\underset{\sigma^{\prime }\in Q}{\sum }\lambda_{\text{abs}}(\sigma^{\prime },\sigma )\\ & +\underset{\sigma^{\prime }\in Q}{\sum }\lambda_{\text{abs}}(\sigma ,\sigma^{\prime })P_{\text{abs}}(\sigma^{\prime },t) \end{array}$$

where  represents the set of all the possible *N* relative configurations of motor-proteins. Thus, the Master equation does hold in terms of the transition probabilities and this implies that the underlying model that governs the dynamics of relative configurations is indeed Markov.

By enumerating the strings *σ*_1_,...,*σ*_*N*_ that represent relative configurations, we let *P*_1_(*t*),...,*P*_*N*_(*t*) represent the probabilities of having the system in each one of the string configurations and define, the probability vector *P*(*t*)=(*P*_1_(*t*),...,*P*_*N*_(*t*))^*T*^. Using the expressions of the transition rates *λ*_*rel*_(*σ*_*j*_,*σ*_*i*_) and Equation (7) it can be shown that

the Markov model that describes the time dynamics of the probability vector *P*(*t*) is given by

(8) $$\begin{array}{l} \frac{d}{\text{dt}}P(t)=\mathrm{AP}(t) \end{array}$$

where $A\in {ℜ}^{N\times N}$ is a sparse stochastic matrix completely determined by the transition rates *λ*_*abs*_(*σ*_*j*_,*σ*_*i*_): if *i*≠*j* then $A_{\text{ji}}=\lambda_{\text{abs}}(\sigma_{j},\sigma_{i})$, otherwise $A_{\text{ii}}=1-\underset{j\neq i}{\sum }\lambda_{\text{abs}}(\sigma_{j},\sigma_{i})$. Starting from an initial probability vector *P*(*t*_0_), it holds that

(9) $$\begin{array}{l} P(t)=\exp(A(t-t_{0}))P(t_{0}) \end{array}$$

where exp(At) is the matrix exponential.

In the specific of kinesin motors, for realistic values of the system parameters and number of motors ($\bar{m}\leq 8$), the dimension of *A* is in the order of 10^5^−10^7^, making the problem of computing exp(A) manageable for a standard desktop computer. For more complex scenarios (i.e. multiple species of motor proteins or larger ensembles) the problem is still tractable using computer clusters or supercomputers

### Determination of biologically relevant quantities

In the previous section, starting from an infinite dimensional model that describes the system dynamics, we have defined a finite dimensional model that keeps track of the relative distances among the motors of the ensemble. The effectiveness of this finite dimensional model is given by the fact that biologically relevant quantities of the system can be computed using explicit formulas without taking recourse to Monte Carlo simulations. Indeed, the probability distribution *P*(*t*) of the different configurations provides detailed information about the system, since it provides the probability associated with every specific relative arrangement of motors on the microtubule. Once the probability of having a certain pattern of motors with all the associated relative distances is known, it is possible to determine many quantities of biological interest for the system. In the following, we provide the expressions of certain biologically relevant quantities, as obtained from our finite dimensional model. They will be considered for the validation of the methodology and in the discussion of novel results.

#### Average number of engaged motors

At any time *t*, the average of the number of engaged motors *m*(*t*) is given by

(10) $$\begin{array}{l} E[m(t)]=\overset{N}{\underset{i=1}{\sum }}M(\sigma_{i})P_{i}(t). \end{array}$$

where *M*(*σ*) represents the number of symbols ’*M*’ in the string *σ*.

#### Average velocity and average runlength

To arrive at the average run-length and average velocity, we will first determine the expected change in the cargo position in a time *Δt* given that the relative configuration changes from *σ* at time *t* to a relative configuration $\sigma^{\prime }$ at time *t*+*Δt*. This expected value can be obtained from the following steps (a) determining the change

$$\begin{array}{l} d(Z^{\prime },Z)=x_{\text{eq}}(Z^{\prime })-x_{\text{eq}}(Z) \end{array}$$

in the cargo equilibrium position for every possible transition from an initial absolute configuration *Z* at time *t* to the final absolute configuration $Z^{\prime }$ at time *t*+*Δt*, where, *Z* and $Z^{\prime }$ have relative configurations *σ* and $\sigma^{\prime }.$ (b) Determine, for every eligible $\left(Z^{\prime },Z\right)$ pair, the probability $P(Z^{\prime },t+\mathrm{\Delta t},Z,t|\sigma^{\prime },t+\mathrm{\Delta t},\sigma ,t)$ of transitioning from $Z\to Z^{\prime }$ conditioned on the specification that relative configuration transitions from *σ* to $\sigma^{\prime }$. (c) Form a weighted sum of $d(Z^{\prime },Z)$ with weights given by probabilities

$$P(Z^{\prime },t+\mathrm{\Delta t},Z,t|\sigma^{\prime },t+\mathrm{\Delta t},\sigma ,t).$$

We first note that the change in the equilibrium position of the cargo is translation invariant. That is if the initial and the final absolute configurations are translated by the same amount then the change in the cargo position remains unaltered. Thus $d(Z^{\prime },Z)=d(\rho^{\alpha }Z^{\prime },\rho^{\alpha }Z)$ for any absolute configurations *Z* and $Z^{\prime }$.

As in the determination of the transition rates *λ*_*rel*_, fix two arbitrary absolute configurations *Z* and $Z^{\prime }$ such that *Π*^(*e*)^(*Z*)=*σ* and $\Pi^{\left(e\right)}(Z^{\prime })=\sigma^{\prime }$. The expected change in the cargo position when the relative initial and final configurations at *t* and *t*+*Δt* are restricted to be *σ* and $\sigma^{\prime }$ respectively is given by

$$\begin{array}{ll} \;d_{\text{av}}(\sigma^{\prime },\sigma ) & :=\;\;\underset{K^{\prime }\in Z(\sigma^{\prime })}{\sum }\underset{K\in Z(\sigma )}{\sum }d(K^{\prime },K)P(K^{\prime },t+\mathrm{\Delta t},K,t|\sigma^{\prime },t\\ & \;+\mathrm{\Delta t},\sigma ,t)\\ & =\underset{\alpha }{\sum }\underset{\beta }{\sum }d(\rho^{\beta }Z^{\prime },\rho^{\alpha }Z)P(\rho^{\beta }Z^{\prime },t+\mathrm{\Delta t},\rho^{\alpha }Z,t|\sigma^{\prime },t\\ & \;+\mathrm{\Delta t},\sigma ,t)\\ & =\underset{\alpha }{\sum }\underset{\beta }{\sum }d(\rho^{\beta }Z^{\prime },\rho^{\alpha }Z)\\ & \;\times \frac{P(\rho^{\beta }Z^{\prime },t+\mathrm{\Delta t},\rho^{\alpha }Z,t,\sigma^{\prime },t+\mathrm{\Delta t},\sigma ,t)}{P_{\text{rel}}(\sigma^{\prime },t+\mathrm{\Delta t},\sigma ,t)}\\ & =\underset{\alpha }{\sum }\underset{\beta }{\sum }d(\rho^{\beta }Z^{\prime },\rho^{\alpha }Z)\frac{P_{\text{abs}}(\rho^{\beta }Z^{\prime },t+\mathrm{\Delta t},\rho^{\alpha }Z,t)}{P_{\sigma }(\sigma^{\prime },t+\mathrm{\Delta t},\sigma ,t)}\\ & =\underset{\alpha }{\sum }\underset{\beta }{\sum }d(\rho^{\beta }Z^{\prime },\rho^{\alpha }Z)\frac{\lambda_{\text{abs}}(\rho^{\beta }Z^{\prime },\rho^{\alpha }Z)P_{Z}(\rho^{\alpha }Z,t)}{\lambda_{\text{rel}}(\sigma^{\prime },\sigma )P_{\text{rel}}(\sigma ,t)}\\ & =\frac{1}{\lambda_{\text{rel}}(\sigma^{\prime },\sigma )P_{\text{rel}}(\sigma ,t)}\underset{\alpha }{\sum }P_{\text{abs}}(\rho^{\alpha }Z,t)\\ & \;\times \underset{\beta }{\sum }d(\rho^{\beta }Z^{\prime },\rho^{\alpha }Z)\lambda_{\text{abs}}(\rho^{\beta }Z^{\prime },\rho^{\alpha }Z)\\ & =\frac{1}{\lambda_{\text{rel}}(\sigma^{\prime },\sigma )P_{\text{rel}}(\sigma ,t)}\underset{\alpha }{\sum }P_{\text{abs}}(\rho^{\alpha }Z,t)\\ & \;\times \underset{\beta }{\sum }d(\rho^{\beta -\alpha }Z^{\prime },Z)\lambda_{\text{abs}}(\rho^{\beta -\alpha }Z^{\prime },Z)\\ & =\frac{1}{\lambda_{\text{rel}}(\sigma^{\prime },\sigma )P_{\text{rel}}(\sigma ,t)}\underset{\alpha }{\sum }P_{\text{abs}}(\rho^{\alpha }Z,t)\\ & \;\times \underset{\beta }{\sum }d(\rho^{\beta }Z^{\prime },Z)\lambda_{\text{abs}}(\rho^{\beta }Z^{\prime },Z)\\ & =\frac{1}{\lambda_{\text{rel}}(\sigma^{\prime },\sigma )}\underset{\beta }{\sum }d(\rho^{\beta }Z^{\prime },Z)\lambda_{\text{abs}}(\rho^{\beta }Z^{\prime },Z)\\ & =\frac{1}{\lambda_{\text{rel}}(\sigma^{\prime },\sigma )}\underset{K^{\prime }\in Z(\sigma^{\prime })}{\sum }d(K,Z)\lambda_{\text{abs}}(K^{\prime },Z) \end{array}$$

where the above equalities follow using similar arguments utilized in deriving relations in (5). We observe that the result is identical for all configurations *Z* such that *Π*^(*e*)^(*Z*)=*σ*. Thus, we can write

$$\begin{array}{l} d_{\text{av}}(\sigma^{\prime },\sigma )=\underset{K\in Z(\sigma )}{\min}\underset{K^{\prime }\in Z(\sigma^{\prime })}{\sum }d(K,Z)\lambda_{\text{abs}}(K^{\prime },K) \end{array}$$

in order to obtain a right hand side that is formally a function of *σ* and $\sigma^{\prime }$ only.

Once the expected value $d_{\text{av}}(\sigma^{\prime },\sigma )$ of the change in cargo position in a time *Δt* when the transitions are restricted to have relative configuration *σ* at time *t* and $\sigma^{\prime }$ at *t*+*Δt* respectively, is found, the expected change in cargo position in a time *Δt* can be determined via

$$\begin{array}{ll} \Delta_{\mathrm{\Delta t}} & :=\underset{\sigma \in Q}{\sum }\underset{\sigma^{\prime }\in Q}{\sum }d_{\text{av}}(\sigma^{\prime },\sigma )P_{\text{rel}}(\sigma^{\prime },t+\mathrm{\Delta t},\sigma ,t)\\ & :=\underset{\sigma \in Q}{\sum }\underset{\sigma^{\prime }\in Q}{\sum }d_{\text{av}}(\sigma^{\prime },\sigma )\lambda_{\text{rel}}(\sigma^{\prime },\sigma )\mathrm{\Delta t}P_{\text{rel}}(\sigma ,t). \end{array}$$

Thus the average velocity is found to be

$$v(t)=\Delta_{\mathrm{\Delta t}}/\mathrm{\Delta t}=\underset{\sigma \in Q}{\sum }\underset{\sigma^{\prime }\in Q}{\sum }d_{\text{av}}(\sigma^{\prime },\sigma )\lambda_{\text{rel}}(\sigma^{\prime },\sigma )P_{\text{rel}}(\sigma ,t).$$

An important quantity that can be experimentally measured in experiments is the expected run-length of the motors, that is the average length traveled by the cargo/motor complex before movement is arrested or the motor detaches from the microtubule lattice. The average run-length can be determined from the knowledge of the probability vector *P*(*t*) on relative configurations.

Then, the average length is given by

$$\begin{array}{ll} \;\text{Average}\;\text{Runlength} & =\int_{0}^{+\infty }v(t)\text{dt}\\ & =\int_{0}^{+\infty }\underset{\sigma \in Q}{\sum }\underset{\sigma^{\prime }\in Q}{\sum }d_{\text{av}}(\sigma^{\prime },\sigma )\lambda_{\text{rel}}(\sigma^{\prime },\sigma )\\ & \;P_{\text{rel}}(\sigma ,t)\mathrm{dt.} \end{array}$$

#### Distribution of step length

The knowledge of the probability of the relative configurations allows one to determine the distribution of the length of the steps observed in the cargo motion. Let *g*(*Z*,*l*) be the set of all absolute configurations such that, if $Z^{\prime }\in g(Z,l)$, $x_{\text{eq}}(Z^{\prime })-x_{\text{eq}}(Z)=l$. Then, the probability rate *μ*^(*l*,*Z*)^(*l*,*Z*) of having a step of length *l* given the absolute configuration *Z* is

(11) $$\begin{array}{l} \mu^{\left(l,Z\right)}(l,Z)=\underset{Z^{\prime }\in g(Z,l)}{\sum }\lambda_{\text{abs}}(Z^{\prime },Z)P_{\text{abs}}(Z,t). \end{array}$$

By summing over all the shifted configurations of *Z* we obtain the probability rate *μ*^(*l*,*σ*,*t*)^(*l*,*σ*,*t*) of having a step of length *l* given the relative configuration *σ*=*Π*^(*e*)^(*Z*) at time *t*

(12) $$\begin{array}{ll} \;\mu^{\left(l,\sigma ,t\right)}(l,\sigma ,t) & \;=\;\overset{+\infty }{\underset{\alpha =-\infty }{\sum }}\underset{Z^{\prime }\in g(\rho^{\alpha }Z,l)}{\sum }\lambda_{\text{abs}}(Z^{\prime },\rho^{\alpha }Z)P_{\text{abs}}(\rho^{\alpha }Z,t)\; \end{array}$$

(13) $$\begin{array}{lll} & =\overset{+\infty }{\underset{\alpha =-\infty }{\sum }}\underset{\rho^{\alpha }Z^{\prime }\in g(\rho^{\alpha }Z,l)}{\sum }\lambda_{\text{abs}}(\rho^{\alpha }Z^{\prime },\rho^{\alpha }Z)\; & \;\\ & \;\times P_{\text{abs}}(\rho^{\alpha }Z,t)\; \end{array}$$

(14) $$\begin{array}{ll} & =\overset{+\infty }{\underset{\alpha =-\infty }{\sum }}\underset{Z^{\prime }\in g(Z,l)}{\sum }\lambda_{\text{abs}}(Z^{\prime },Z)P_{\text{abs}}(\rho^{\alpha }Z,t)\; \end{array}$$

(15) $$\begin{array}{ll} & =P_{\text{rel}}(\sigma ,t)\underset{Z^{\prime }\in g(Z,l)}{\sum }\lambda_{\text{abs}}(Z^{\prime },Z).\; \end{array}$$

As a consequence, summing over all the relative configurations *σ*_1_,...,*σ*_*N*_ allows one to obtain the probability rate *μ*^(*l*,*t*)^(*l*,*t*) of a step of length *l* at time *t*

(16) $$\begin{array}{l} \mu^{\left(l,t\right)}(l,t)=\overset{N}{\underset{i=1}{\sum }}P(\sigma_{i},t)\underset{Z^{\prime }\in g(Z,l)}{\sum }\lambda_{\text{abs}}(Z^{\prime },Z). \end{array}$$

Since the probability rate of the length of a step is proportional to its frequency, the probability *P*^(*l*,*t*)^(*l*,*t*) of a step of size *l* at time *t* is

(17) $$\begin{array}{l} P^{\left(l,t\right)}(l,t)=\frac{\mu^{\left(l,t\right)}(l,t)}{\underset{x\in \chi }{\sum }\mu^{\left(x,t\right)}(x,t)} \end{array}$$

where *χ*={*x*:*μ*^(*x*,*t*)^(*x*,*t*)≠0}. This formula provides an exact computation of the distribution of the step size from the model parameters without relying on histograms obtained from Monte Carlo simulations.

## Results and discussions

### Methodology validation

While the methods developed in this article can be applied to study ensembles of motor proteins of any class, validation will be presented on kinesin motors. We first derive the transition rates *λ*_*abs*_ between absolute configurations for an ensemble of kinesin motors.

#### Obtaining transition rates on the absolute configuration space

The determination of transition rates are based on experimental data and theoretical considerations, where, rates from single-motor experiments are used to derive transition rates in the case where an ensemble of motors are involved in transport. Most of the modeling assumption are the same as made in [7] with minor differences which are described next.

### Probability of stepping under a force *F* for kinesin

During a step a protein *M* converts ATP into kinetic energy and ADP

(18) $$M+\text{ATP}\overset{k_{\text{on}}}{\underset{k_{\text{off}}}{⇄}}M\;\text{ATP}\overset{k_{\text{cat}}}{\to }M+\text{ADP}+P_{i}+\mathrm{energy.}$$

Following [11], Michaelis-Menten dynamics predicts a ATP hydrolysis rate equal to *k*_*cat*_[*ATP*]/([*ATP*]+*k*_*m*_), where *k*_*m*_=(*k*_*cat*_+*k*_*off*_)/*k*_*on*_. In addition, the free head of the motor is assumed to bind to the microtubule location with a defined probability (or efficiency) *ε*. In this scenario, the probability *P*_*step*_ of stepping

for a single motor is given by

(19) $$\begin{array}{ll} & P_{\text{step}}=\frac{k_{\text{cat}}[\;\text{ATP}]}{[\;\text{ATP}]+k_{m}}\mathrm{\varepsilon .}\; \end{array}$$

The force *F* that the cargo exerts on the motor is assumed positive when it opposes the motor motion. When the force exceeds the stalling force *F*_*s*_, it causes the motor to stall. Following [7], the force *F* is assumed to affect the motor dynamics by changing the probability *ε* of binding to the microtubule, following the relation

(20) $$\begin{array}{l} \varepsilon (F)=\left\{\begin{array}{cc} 1 & \;\text{if}F\leq 0\\ 1-{\left(\frac{F}{F_{s}}\right)}^{2} & \text{if}0<F<F_{s}\\ 0 & \;\text{otherwise}. \end{array}\right) \end{array}$$

In [7] it is assumed that the force *F* also influences the kinetics of the ATP hydrolysis. In particular it is assumed that *k*_*off*_ increases with increasing *F* according to the relation $k_{\text{off}}=k_{0\text{off}}e^{{\text{Fd}}_{l}/K_{b}T}$, where *k*_0*o* *f* *f*_ is the backward reaction rate of the hydrolysis when *F*=0, *K*_*b*_ is the Boltzmann constant, *T* is the temperature and *d*_*l*_ is a parameter that can be experimentally determined. Thus, the transition rate for a step under a constant force *F* is given by

(21) $$\begin{array}{ll} P_{\text{step}}(F) & =\frac{k_{\text{cat}}[\text{ATP}]}{[\text{ATP}]+\frac{k_{\text{on}}+k_{\text{off}}(F)}{k_{\text{cat}}}}\varepsilon (F).\; \end{array}$$

Under the assumption that the cargo position follows a truncated Gaussian distribution with probability density, for |*x*|<3*σ*_*th*_,

(22) $$\begin{array}{l} \phi (x)=\left(e^{-\frac{x^{2}}{2\sigma_{\text{th}}^{2}}}\right)/\left(2\int_{0}^{3\sigma_{\text{th}}}e^{-\frac{x^{2}}{2\sigma_{\text{th}}^{2}}}\text{dx}\right), \end{array}$$

the transition rate is determined averaging over the position of the cargo

(23) $$\begin{array}{ll} \lambda_{\text{abs}}(Z,Z+R_{k}^{\left(\text{step}\right)})= & z_{k}\int_{x_{\text{eq}}(Z)-3\sigma_{\text{th}}}^{x_{\text{eq}}(Z)+3\sigma_{\text{th}}}P_{\text{step}}(F(x-a_{k}))\\ & \phi (x-x_{\text{eq}}(Z))\text{dx} \end{array}$$

where the term *z*_*k*_ represents the number of motors in the *k*-th location (the transition rate is proportional to the number of motors in the location) and the term *a*_*k*_ is the position of the *k*-th location.

### Probability of detachment

From [Schnitzer et al. 2000], the processivity *L* is

(24) $$\begin{array}{l} L=\frac{d_{s}[\text{ATP}]Ae^{-F\delta_{l}/K_{b}T}}{[\text{ATP}]+B(1+A)e^{-F\delta_{l}/K_{b}T}}, \end{array}$$

where *A*, *B* and *δ*_*l*_ are again parameters that can be experimentally determined. Since the processivity represents how far a motor can move, on average, before detaching from the microtubule, we find a relation between the probability of stepping and the probability of detachment.

(25) $$\begin{array}{l} \frac{P_{\text{step}}(F)}{P_{\text{detach}}(F)}=\frac{L}{d_{s}}=\frac{[\text{ATP}]Ae^{-F\delta_{l}/K_{b}T}}{[\text{ATP}]+B(1+A)e^{-F\delta_{l}/K_{b}T}}. \end{array}$$

Thus, so long as *F*<*F*_*s*_,

(26) $$\begin{array}{l} P_{\text{detach}}(F)=\frac{[\text{ATP}]+B(1+A)e^{-F\delta_{l}/K_{b}T}}{[\text{ATP}]Ae^{-F\delta_{l}/K_{b}T}}P_{\text{step}}(F). \end{array}$$

When *F*≥*F*_*s*_, in [7] a constant detachment rate is assumed *P*_*detach*_(*F*)=*P*_*back*_=2*s*^−1^. Analogously to the previous case, the transition rate associated to the detachment event is

(27) $$\lambda_{\text{abs}}(Z,Z-R_{k}^{\left(\text{att}\right)})=$$

(28) $$z_{k}\int_{x_{\text{eq}}(Z)-3\sigma_{\text{th}}}^{x_{\text{eq}}(Z)+3\sigma_{\text{th}}}P_{\text{detach}}(F(x-a_{k}))\phi (x-x_{\text{eq}}(Z))\mathrm{dx.}$$

### Probability of attachment

Experimentally, it is found in [21,22] that the probability of a kinesin motor attaching to the microtubule is *P*_*att*_≃5*s*^−1^. If the motor is linked to the cargo, it is assumed that it attaches to the microtubule without stretching its linkage. Thus, the only admissible locations of attachment are the locations at a distance from the cargo that is less than *l*_0_. They are also assumed all equally likely.

### Numerical parameters

The numerical parameters that we have considered in our analysis of Kinesin-I ensembles, when not otherwise specified, are *k*_*cat*_=105 *s*^−1^, *k*_*on*_=2·10^6^*M*^−1^*s*^−1^, *k*_0*o* *f* *f*_=55 *s*^−1^, [*ATP*]=2·10^−3^*M*, *F*_*s*_=0.006 *nN*, *d*_*s*_=8 *nm*, *d*_*l*_=1.6 *nm*, *δ*_*l*_=1.3 *nm*, *A*=107, *B*=0.029 *μM*, *T*=300*Kk*_*el*_=0.32·10^−3^*nN*/*nm*. All these parameters are the same used in [7] and have been experimentally determined.

Using these parameters an upper-bound on *s*^(*m* *a* *x*)^ (see Equation (3)) on the extent of any relative configuration is found to be 320 *nm*, for ensemble of at most 4 motors. This extent is rather large given that the length of a Kinesin molecule is in the hundreds of nanometer range. We remark that the *s*^(*m* *a* *x*)^ is an upper bound on the possible extent. Thus there are avenues to be explored where a smaller extent can be assumed. We enumerated the finite number of relative configurations as *σ*_1_,…,*σ*_*N*_ and determined transition rates *λ*_*r* *e* *l*_(*σ*_1_,*σ*_2_), from a relative configuration *σ*_1_ to another relative configuration *σ*_2_, using Equation (6).

As in [7], we have considered ensembles of at most four motors, and, we computed the probability vector *P*(*t*) exactly. We remark that *P*(*t*) depends on the initial probability *P*(*t*_0_), as shown in Equation (9). In all our computations for validation purposes we have assumed the same initial probability distribution *P*(*t*_0_) that is used in [7].

#### Validation of the average velocity and average run-length

*Average Runlength:* In [7], in one scenario (Model A) the authors neglect the effect of thermal noise, and in another scenario (Model B) they introduce a dynamic model for the Brownian motion of the cargo. We have performed the same kind of separated analysis following our approach. First, we have fixed the variance of the cargo position *σ*_*th*_ to zero, making our model analogous to “Model A” in [7].

For the initial distribution we consider that at time *t*=−1 *sec* exactly one motor is attached to the microtubule and that the cargo is not being lost before time *t*=0. In the time interval [−1 *sec*,0 *sec*] the motors behave as usual. The probability distribution of their configurations at time *t*=0 is the initial probability distribution for all our simulations. This initialization is similar to the one described in [7].

**Figure 5:**
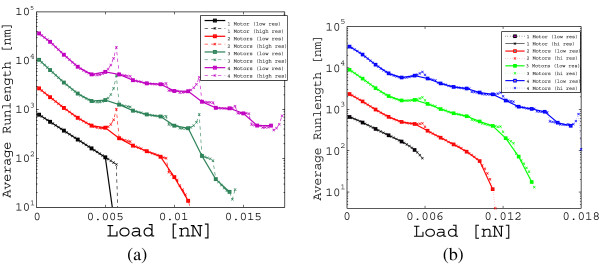
**Average run-length as a function of the load applied to the cargo neglecting the thermal noise component (a) and considering it (b).** The solid lines have been plotted for loads that are multiple values of 0.001 *nN* and agree with the Monte Carlo simulations in [7] using the same set of parameters. The dashed lines are the same plots for loads that are multiple values of 0.0002 *nN*. Observe the presence of peaks that were unnoticed at the previous resolution.

The results of this noiseless analysis are reported in Figure 5 using both a coarse grid (solid lines) and a fine grid (dashed lines) for the load force. The coarse grid has a resolution of 1 *pN*, exactly as for the run-length curves computed in [7], while for the finer grid we have chosen a resolution of 0.2 *pN*.

We find a practically exact quantitative agreement of our exact results and the one based on Monte Carlo simulations as presented in [7] which correspond to a coarse grid resolution of 1 pN, when there is no noise (compare Figure 5(a) with Figure 5(a) in [7]). In particular, for all possible sizes of the ensembles, we find run-length curves that are monotonically decreasing with higher loads. In a similar manner a near quantitative agreement is found when noise is present (compare Figure 5 with Figure 5(b) in [7]).

**Figure 6:**
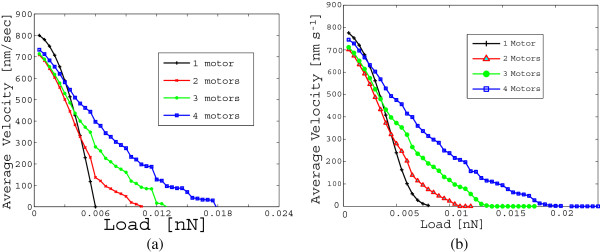
**Average velocity of the cargo as a function of the load in the case of ensemble of motors of different sizes (from one motor to four motors) in a case where thermal fluctuations are neglected (a) and where they are approximated with a truncated Gaussian (b).** These curves, obtained via our exact method, reproduce the Monte Carlo simulation results in [7] for the same set of parameters.

Under the condition that the cargo is not lost, a steady state probability distribution will be reached. The corresponding vector of probabilities *Π* can be used to determine the average velocity of the cargo when at least one motor is engaged on the microtubule. Results in Figure 6(a) are based on the noiseless scenario equivalent to Model A in [7]. Analogous results are reported in Figure 6(b) for the noisy scenario. Our results match with the results obtained in [7] using Monte Carlo simulations (see Figure 3(a) and Figure 3(b) in [7]). Indeed, one of the findings in [7] was that at low loads a cargo carried by one single motor moves faster than a cargo carried by more motors. The main difference is that the results obtained using our probabilistic method are exact and based on a precise definition of steady state. Conversely, in [7] certain approximations are required (i.e. a maximal duration for the transient is assumed) and the accuracy depends on the number of simulations performed.

### Discussion

The methodology developed in this article allows one to determine how the probabilities of different relative arrangements of molecular motors on a microtubule change over time.

This information is contained in the probability vector *P*(*t*) (see Equation 9). For physical values of the model, the number *N* of arrangements is limited and allows its direct computation. The knowledge of *P*(*t*) offers, from a biological perspective, detailed information about the system. In fact, except for the absolute position of the motor ensemble on the microtubule (that is lost in the string representation), the information about the system is completely preserved. When *P*(*t*) is known, it is possible to determine, via explicit formulas, many quantities of biological interest, such as the average run-length of the ensemble, the average number of engaged or active motors, the average instantaneous velocity at which the cargo moves and the probability distribution of the step sizes observed in the cargo motion. Contrary to other methods, the final accuracy of the results does not depend on any specific simulation technique or on the number of stochastic simulations that are performed. Also, the method is extremely efficient: even for practically sized ensembles with $\left(\bar{m}\leq 8\right)$, results can be computed on a standard desktop computer and general purpose software. For a large range of physically meaningful values of the parameters, the number *N* of possible string configurations is in the order of about 10^5^−10^7^. Furthermore, the matrix , that defines the dynamics of the ordinary differential equation to be solved, is sparsely populated. The manageable dimension of the system state and the high sparsity of  make the computation of the exponential of  feasible even with a limited amount of memory. As evidence for this, all the results shown in this article have been obtained using a machine equipped with a quadcore processor and 4 *Gb* memory (the algorithm was implemented using MATLAB, TheMathWorks, Natick, MA). For the study of larger groups of motor proteins, the adoption of computer clusters or super-computers still remains a viable solution.

#### Presence of a steady state

The probability vector *P*(*t*) for the different arrangements of motors of the ensemble depends on the initial probability *P*(*t*_0_), as shown in Equation (9). A fundamental question is whether starting from any arbitrary initial condition, after a transient period, the motor ensemble eventually behaves according to a fixed probability distribution which does not depend on the initial distribution of motors. A property of this kind would justify the experimentally observed robustness of the system. Furthermore, it would make it possible to determine the generality of certain observations, independent from the initial distribution *P*(*t*_0_).

**Figure 7:**
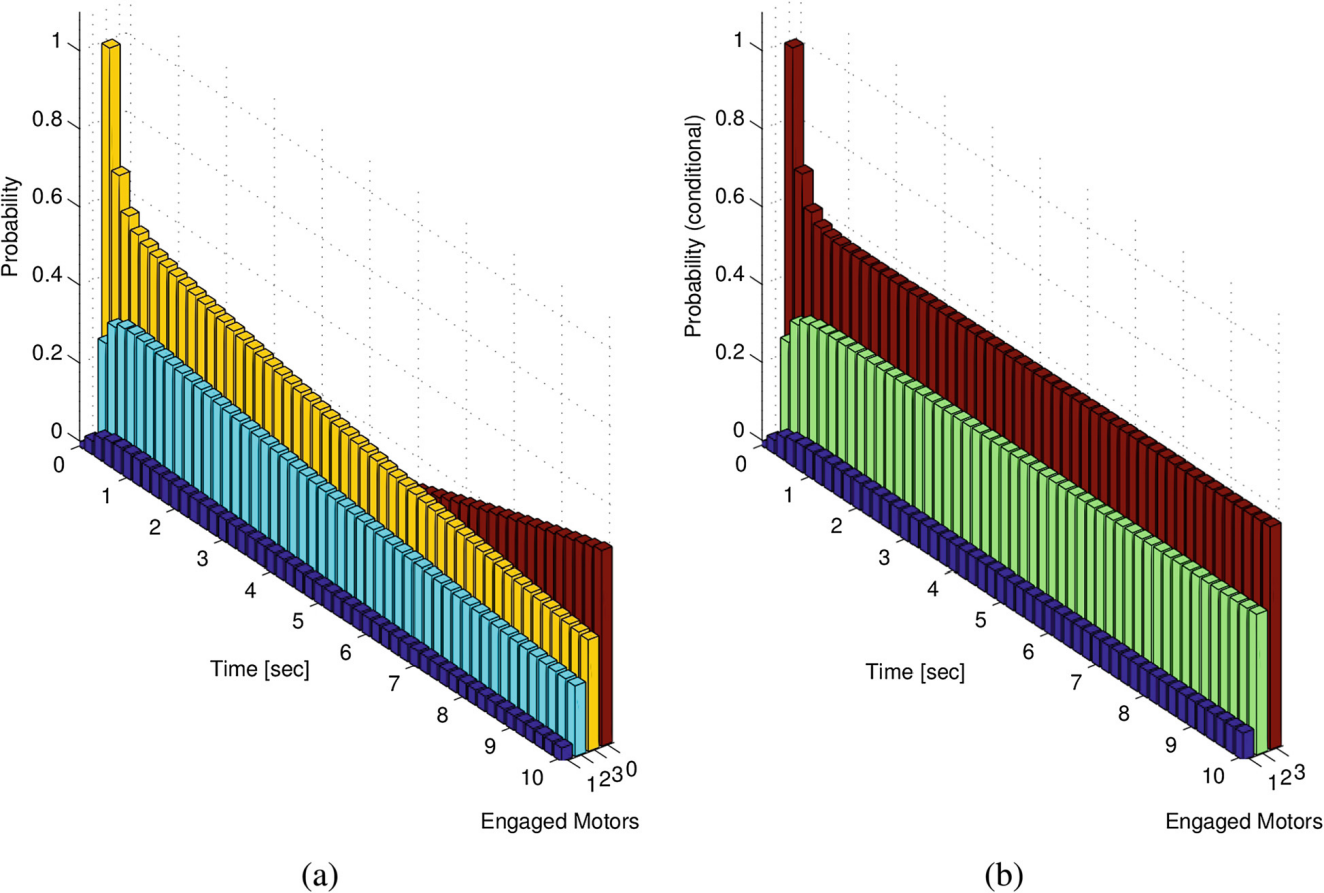
**Probability of having 1, 2, 3 or 0 motors engaged on the microtubule as a function of time *t * (a).** Probability of having 1, 2, 3 motors engaged on the microtubule as a function of time *t* under the condition that there is at least one motor engaged **(b)**. The probability of having no engaged motors converges to 1 as time goes to infinity. Observe, instead, that a constant probability distribution is reached in case **(b)** where it is assumed that at least one motor is engaged.

In order to illustrate how to define a meaningful notion of steady state, it is useful to start considering how the probability distribution of the number of motors engaged on the microtubule changes over time. In Figure 7(a), the knowledge of *P*(*t*) is used to determine the probability of having a given number of motors engaged on the microtubule at time *t*, assuming an ensemble of three motor proteins ($\bar{m}=3$).

The probability of having no engaged motors on the microtubule slowly converges to 1 as *t* goes to infinity. This corresponds to an intuitive fact: the loss of the cargo is an irreversible event for the system and, sooner or later, it is to be expected that all motors will detach from the microtubule. According to the model formulation, the loss of the cargo is always the final event and, as such, it trivially represents the only steady state condition of the system. However a non-trivial, and biologically more meaningful, notion of “steady state” can be introduced. Figure 7(b) shows the conditional probability distribution of the number of motors engaged on the microtubule at time *t*, *given that at least one motor is engaged*. This conditional probability distribution converges to a non-trivial distribution. Thus, under the assumption that the cargo has not been lost, the number of motors reaches a probability distribution that does not depend on its initial condition. This holds not only for the probability distribution of the number of engaged motors, but, more generally for the probability distribution of relative configurations, as we will show at the end of this section.

In other words, under the hypothesis that the cargo has not been lost, the relative arrangements of the motors on the microtubule reach a stable (conditional) probability distribution $\Pi \in {ℜ}^{N-1}$. The determination of *Π*, once *P*(*t*) is known, is quite straightforward and can be obtained directly from the definition of conditional probability. The knowledge of this steady state *Π* provides key insights into the behavior of a group of motors.

**Figure 8:**
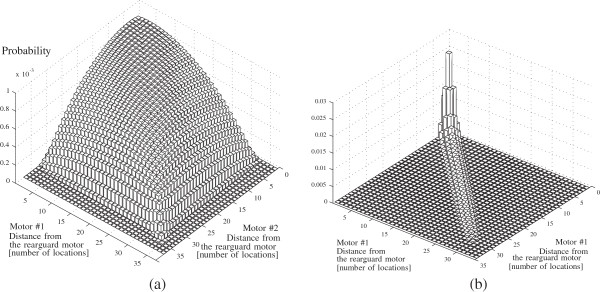
**A representation of the steady state probability distributions *Π * for an ensemble of three motors with low load (a) and high load on the cargo (b).** The rear-guard motor is taken as a reference. The *x* and the *y* axes represent the distance of the other two motors from the rear-guard one. The distance is expressed in number of locations on the microtubule. The *z* axis represent the probability associated with a specific relative configuration. In the case of a low load, the motor proteins tend to be more spread out. Instead, in the case of a high load we observe that they tend to be clustered together or to prefer a configuration with two leading motors and the rear-guard lagging behind.

For $\bar{m}=3$, we have computed the steady state conditional probability *Π* of the motor arrangements in two different cases: cargo subject to low load (*F*_*load*_=0.0002*nN*) and cargo subject to high load (*F*_*load*_=0.008*nN*). The results are depicted in Figure 8(a) and in Figure 8(b), respectively.

Data in Figure 8(a-b) provide the following insight. The rearguard motor is always assumed as a reference and the *x*-*y* axes represent the relative distances of the first and second motors of the ensemble from the rearguard one. Thus, each point *x*-*y* represents an arrangement of the ensemble. On the *z* axis we report the probability of that specific arrangement. The probabilities of configurations with less than three motors engaged are not reported in the two figures because that would make the visualization difficult. What is important to notice is how the presence of either a low load or a high load leads to two different steady state situations. Thus under a low load the motors tend to spread out almost uniformly, instead, under a high load, a certain pattern of configurations emerge as being more likely. The most likely configurations lie along the diagonal *x*=*y*, with a prominent peak around the origin *x*=*y*=0. This means that, under a high load, eventually it is more likely to find all the three motors clustered together (represented by the peak at the origin). The high frequency of configurations along the *x*=*y* diagonal suggests that it also likely to find two close leading motors with the third one lagging behind. Observations like this would be difficult to obtain using Monte Carlo simulations. Instead, an exact computation of the probabilities allows to infer these characteristics of the motion in a comprehensive manner.

#### Existence and uniqueness of the conditional stationary distribution

In this section we provide the proof that there is a unique non-trivial (conditional) stationary distribution for the relative configurations *σ*_1_,...,*σ*_*N*_ under the assumption that there is at least one motot attached to the microtubule. Without any loss of generality assume that *σ*_*N*_=*∅* is the state associated with the loss of the cargo and that *σ*_1_=^′^*M*^′^ is the state associated with exactly one motor attached to the microtubule. Observe that in graph associated with this Markov system all state *σ*_2_,...,*σ*_*N*−1_ can reach *σ*_1_ via a sequence of detachments. Again without any loss of generality, let us reorder the states assuming that the first *N*_*a*_, $\sigma_{2},\mathrm{...},\sigma_{N_{a}}$, can be reached from *σ*_1_ in the graph associated with the Markov model and that the states $\sigma_{N_{a}+1},\mathrm{...},\sigma_{N-1}$ can not be reach from *σ*_1_. The uniqueness of the stationary distribution is equivalent to showing that there is a unique vector (*Π*_1_,...,*Π*_*N*−1_)^*T*^ such that $\overset{N-1}{\underset{j=1}{\sum }}\Pi_{j}=1$ and 

where 

• each column of the matrix sums up to zero

• the upper triangular structure of the matrix derives from the particular way we have reordered the states in accessible from *σ*_1_ and not accessible from *σ*_1_

• the top left entry *A*_1,1_+*A*_*N*,1_ derives from having removed the state *σ*_*N*_=*∅* since we are looking for the conditional distribution of the relative configurations given that the cargo has not been lost.

The bottom right block *A*^(22)^ is such that (*A*^(22)^)^*N*−1^ is a strictly diagonally dominant matrix, since it is possible to reach *σ*_1_ from each of the states $\sigma_{N_{a}+1},\ldots ,\sigma_{N-1}$. This implies necessarily that $\Pi_{N_{a}+1}=\Pi_{N_{a}+2}=\ldots =\Pi_{N-1}=0$. Instead, the top left block of the matrix is an irreducible matrix (in the associated graph each state can reach any other one passing through *σ*_1_) implying that the elements $\Pi_{1},\Pi_{2},\ldots ,\Pi_{N_{a}}$ and are uniquely determined and strictly positive. Thus, there is a unique stationary conditional distribution given that at least one protein is attached to the microtubule.

#### Enabling finer analysis

As our method yields an exact probability distribution, it facilitates a finer analysis. For example, the dependence of the average run length on the load, under the presence and absence of thermal noise, is of interest. In [7], Monte Carlo methods yield the dependence, where the average run-length is obtained with respect to load in steps of 1 *pN*. With our method it is straightforward to obtain the exact values at this force resolution. However, we can obtain this dependence at a finer force resolution of 0.2 *pN*. Using the finer scale, we noticed peaks in the run-length curves for $\bar{m}>2$, that are not evident at the coarse resolution of 1 *pN*. These peaks correspond to loads that are multiples of the stalling force *F*_*s*_. These peaks, ascertained by our method provides the following insight. Let us consider the curve corresponding to $\bar{m}=2$ for simplicity. When only one motor is engaged and *F*_*load*_ is close to (but less than) the stalling force *F*_*s*_, the probability of detachment becomes small, as evident from Equation (26). In this condition the loss of the cargo becomes unlikely. Thus, the disengaged motor, on average, has enough time to attach back to the microtubule, catch up with the leading motor and move the cargo a little further leading to a net increase in the run-length. For $\bar{m}>2$, equivalent arguments can be provided and the peaks on the other curves can be similarly explained. This mechanism shows how, in the absence of the Brownian motion of the cargo, the expected run-length tends to increase while the load approaches values that are multiple of the stalling force. When the Brownian motion of the cargo is taken into account (see Figure 5 (b)) the peaks are smoothed down, but do not disappear, thus they represent a robust characteristic of the model. This kind of non-monotonic behavior for the computed average run-length curve can be a drawback of the detachment rate model, when *F* is close to *F*_*s*_. In such a case, our approach can be seen to identify specific inaccuracies of the model. However, it is also possible that the finer analysis indicates a behavior that is exhibited by an ensemble of motors carrying a cargo and the deviation from a monotonic behavior are not artifacts of the model. In such a case, the finer analysis identifies a mechanism of “coordination” among the motors that optimizes the average run-length in situations close to a stalling scenario. Experiments can be designed and conducted in order to determine whether this mechanism is a model artifact or if it is actually occurring in the physical system. Related but different study on the relationship of velocities and run-length is reported in [23].

#### Detection of rare events

**Figure 9:**
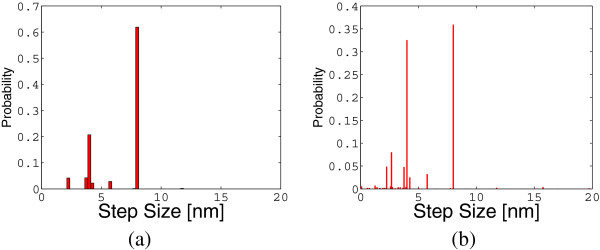
**Computed probabilities for different step sizes for the cargo in the case of an ensemble of two motors (a) and an ensemble of three motors (b).** In the case of two motors, observe the presence of the possibility (with an extremely low probability) of observing steps in the cargo position at about 12 *nm* of length.

The exact determination of the probability distribution of the different configurations allows for the detection of rare events quantifying their probabilities. For example, from *P*(*t*) it is possible to determine the probability of the different steps sizes for an ensemble of 2 motors as represented in Figure 9.

**Figure 10:**
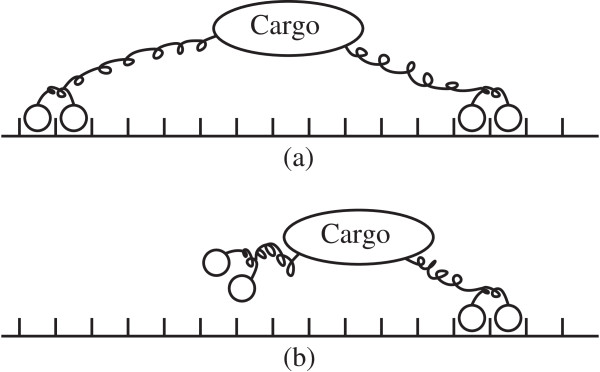
**The mechanism under which steps longer than 8 *n m * can be observed in the cargo motion.** The rearguard motor on the left is actively pulling back the cargo **(a)**. When it detaches from the microtubule, the cargo equilibrium position can advance by more than 8 *nm***(b)**.

We notice two prominent peaks corresponding to 8 *nm* and 4 *nm*. These peaks correspond to the case where there is one active motor before and after the step and to the case where there are two active motors before and after the step, respectively. There are also different predicted step sizes close to 2 *nm*, 6 *nm* and around 4 *nm*. They correspond to situations where there are different number of active motors before and after the step. We also find a small probability of steps larger than 8 *nm* which are closer to 11 *nm*. Since the probability distribution of steps is exactly calculated, there must be events leading to a change in the equilibrium position of the cargo with length longer than 8 *nm*. This is unexpected. Indeed, since each motor can advance only by 8 *nm*, the cargo equilibrium itself can advance by, at most, 8 *nm*. In order to identify the possible causes of these anomalous steps, we have taken into account all the possible transitions from one absolute configuration to another and we have flagged those ones producing “ 11 *nm* steps”. With these exhaustive analysis, we have determined that there are situations where the cargo equilibrium can advance by more than 8 *nm* steps. Indeed, all these situations corresponds to cases where the rearguard motor, which is actively pulling the cargo, detaches from the microtubule. This scenario is schematically represented in Figure 10.

Thus, these “ 11 *nm* steps” are not associated to any actual stepping event of a motor, but exclusively to detachment events of the rearguard motor. This situation is rare in a bead-assay experiment, but it is known that two motors frequently pull the shared microtubule in two opposite directions in a gliding assay experiment (see [8]). Our analysis indicates that a cargo movement with step sizes larger than 8 *nm* is still viable in a bead assay, though infrequent. Our approach can identify the causes for such rarer modalities of transport. Thus, steps larger that 8 *nm* as those described in [8] could well be originated by a mechanism of this kind. The probability distribution of the step size for an ensemble of 3 motors is reported in Figure 9 where the steps longer than 8 *nm* (at about 11 *nm*, 15 *nm* and 20 *nm*) have similar interpretation.

## Conclusions

In conclusion, a framework and model for the study of the coordinated behavior of molecular motors has been introduced. The main novelty of the approach lies in the adoption of methods of analysis that obviate the need of Monte Carlo simulations.

The methodology is applied to the analysis of ensembles of Kinesin-I motors. Results that had been previously found using Monte Carlo methods are accurately reproduced, validating the methodology. More importantly, under this novel probabilistic approach new insights about the mechanisms of action of these proteins are found, suggesting hypothesis about their behavior and driving the design and realization of new experiments. For example, a possible mechanism under which motor proteins could coordinate together in order to increase their overall processivity is identified. Furthermore, the probabilistic framework allows the determination of steady state conditions for groups of molecular motors. The model predicts that, regardless of their initial configuration, the molecular motors will reach a situation where their relative distances on the microtubule will follow the same probability distribution. This provides an explanation for the robustness of the system with respect to the fluctuations of the surrounding environment.

The advantages provided in accuracy and efficiency make it possible to detect rare events in the motor protein dynamics, that could otherwise pass undetected using standard simulation methods. In this respect, the model has allowed to provide a possible explanation for infrequent steps of length longer that 8 *nm* that had been observed in bead assay experiments [8].

## Competing interests

The authors do not have any competing interests in the topics discussed in the article.

## Authors’ contributions

The authors have evenly contributed to the paper results. All authors read and approved the final manuscript.
